# Supplementary material for: Past world economic production constrains current energy demands: Persistent scaling with implications for economic growth and climate change mitigation
Source: PLoS One. 2020 Aug 27;15(8):e0237672. doi: 10.1371/journal.pone.0237672 (PMC7451548; doi:10.1371/journal.pone.0237672)
Supplement: Attachment — Submitted filename: Response to Reviewers.pdf [file pone.0237672.s003.pdf]

Response to Reviewers: Persistent scaling between historically cumulative economic production and current global energy consumption with implications for climate change mitigation

1 Reviewer 1

Reviewer 1: This research is a professionally-written interesting piece to read with credible data sources presenting deep knowledge of the authors. First comment: According to the authors, past production determines the level of energy demand. However, inside the text I have not seen any proof/justification to show the direction of causation. To elaborate more, how one can say that the causation does not run in the opposite direction, i.e., from energy demand to production.

Because the direction of time is from the past to the present, the direction of causation is that past cumulative production determines present energy consumption. Because this is a worthwhile point to emphasize, we have added to the abstract

What this constant implies is that current growth trends in energy consumption, population, and standard of living, perhaps counterintuitively, are a consequence of past innovations that have improved energy efficiency. Predicted growth rates agree well with available historical data.

And in the body of the text

A tree's past seasonal production of leaves contributes to the long term growth of its trunk, even though the leaves die each autumn. It may similarly be that past economic production of all kinds contributed to the cumulative growth of civilization and determines its current energy demands.

Second comment: Another clarification that I would like to see is that according to the authors "there is persistent time-independent scaling between the historical time integral of world inflation-adjusted economic production and current rates of world primary energy consumption". To my understanding, historical time-integral is a time-dependent concept which contradicts the "time-independent" claim by the authors.

Here time-independent refers to the scaling not the historical time integral. We have modified the text in the abstract to read

Drawing from thermodynamic arguments, and using 38 years of available statistics between 1980 to 2017, we find a constant scaling between current rates of world primary energy consumption $\mathcal{E}(t)$ *and the historical time integral* W *of past world inflation-adjusted economic production* Y, *or* $W(t) = \int_0^t Y(t')\,dt'$. *In each year, over a period during which both* $\mathcal{E}$ *and* W *more than doubled, the ratio of the two remained nearly unchanged, that is* $\lambda = \mathcal{E}(t)(t)/W(t) = 5.9 \pm 0.1$ *Gigawatts per trillion 2010 US dollars.*

Last comment: for the model developed by the authors, is it possible to account for other factors such as governmental and/or international intervention policies/strategies towards decarbonization?

Policy measures directed at decarbonization are rather beyond the scope of the paper except where the paper currently reads *For a sense of what this implies, consider that 2.4% of the current global rate of energy consumption* $\mathcal{E} \approx 20$ *TW corresponds to 480 GW. That is to say, for energy consumption to continue to grow at this rate without*

increasing carbon dioxide emissions would require over 1 GW of new power capacity in nuclear or renewable energy to be added online each day, the approximate size of a large central power plant

2 Reviewer 2

Authors of the article "Past production constrains current energy demands: persistent scaling in global energy consumption and implications for climate change mitigation" have selected an interesting topic and developed it well. In my opinion, the paper is qualified to publish in the current form.

No changes made.

3 Addendum

In response to an external comment we added the following statement *It should also be noted that CO_2 uptake is not in fact linear over timescales much longer than decades. The values for* $[\mathrm{CO_2}]_{\mathrm{eq}}$ *presented here do not reflect important non-linearities that might arise from e.g. increasing ocean acidification, and that would allow for concentrations to continue to slowly rise even as energy consumption rates stall [1]. With respect to globally-averaged surface temperature anomalies, it has been argued that they have a linear relationship with cumulative emissions of carbon. This sensitivity depends only weakly upon whether emissions are rising and falling, and the maximum CO_2 concentration that is reached. A value of 1.6 $^\circ C$ per Tt C [2] can be used as a rough guide, in which case persistence of current 10 Gt C yr^{-1} emissions rates would imply a further temperature rise of about 0.5 $^\circ C$ by 2050. Assuming persistence in current 2.4% yr^{-1} energy consumption growth rates, and no further decarbonization, the increase is 0.7 $^\circ C$.*

References

1. Glotter M, Pierrehumbert R, Elliott J, Matteson N, Moyer E. A simple carbon cycle representation for economic and policy analyses. Climatic Change. 2014;126(3-4):319–335. doi:10.1007/s10584-014-1224-y.

2. Zickfeld K, MacDougall AH, Matthews HD. On the proportionality between global temperature change and cumulative CO 2 emissions during periods of net negative CO 2 emissions. Environmental Research Letters. 2016;11(5):055006. doi:10.1088/1748-9326/11/5/055006.
